# A Process Framework of Family AI Use in Early Education and Care

**DOI:** 10.1111/famp.70174

**Published:** 2026-06-16

**Authors:** Ying Zhang, Qilong Zhang, Ke Jiang, Ying Li, Qiuzhi Xie

**Affiliations:** ^1^ College of Education United Arab Emirates University Al Ain UAE; ^2^ Faculty of Art and Education The University of Auckland Auckland New Zealand; ^3^ Australian Centre for the Advancement of Literacy Australian Catholic University Sydney Australia

**Keywords:** artificial intelligence, early childhood, intended function, parental belief, parental mediation

## Abstract

Artificial intelligence (AI) is rapidly entering family life, reshaping how young children learn, play, and interact with technology. Yet little is known about how parents interpret these changes or organize AI use at home. This qualitative study generates a process framework of family AI use in early education and care, capturing Chinese parents' perspectives through inductive thematic analysis. Sixteen parents whose children attended nurseries and kindergartens across nine Chinese cities were purposefully selected for in‐depth interviews. Through inductive analysis, we constructed a process framework suggesting that parents first articulate beliefs about AI, which inform the functions they intend AI to serve, and these functions in turn shape their mediation strategies. Parents emphasized both the significance of AI and the irreplaceability of human roles, while envisioning AI as a tool for relieving workload, offering parenting advice, entertaining, and supporting playful learning. Mediation strategies reflected ongoing negotiation, including managing AI's presence, interface, exposure, screen use, and content. This process framework illustrates how families balance opportunity with caution by shaping children's encounters with AI in ways consistent with parental values. It offers a conceptual tool for researchers, practitioners, and policymakers concerned with supporting healthy family engagement with AI in the digital age.

## 1 Introduction

From storytime apps to AI‐powered learning companions, AI technologies are becoming increasingly embedded in everyday family life. Artificial intelligence (AI) is increasingly shaping how young children explore, learn, and interact at home (Livingstone and Helsper 2008; Nathanson 1999). While research has often focused on AI in classrooms, little attention has been paid to the domestic setting, where parents are the primary guides of early learning. Parents' beliefs about AI, the purposes they intend it to serve, and the ways they supervise or support its use are critical in shaping children's experiences; yet empirical evidence on these factors remains limited. This study investigates how Chinese parents perceive and manage AI use with their young children in the home. Drawing on in‐depth interviews with Chinese parents, the study illuminates how family practices influence AI integration, offering insights for families, educators, and practitioners to support meaningful and developmentally appropriate AI engagement at home.

In this study, we adopt an ecologically grounded definition of AI as digital systems that perceive, process, and respond in human‐like ways (Russell and Norvig 2021). The technologies referenced in this study—voice assistants (Doubao, Tmall Genie), AI learning apps (Zebra Literacy, Hongen Literacy), and smart devices—are predominantly powered by large language models (LLMs). In everyday discourse, parents often used the term “AI” to describe systems that respond interactively to children's inputs (cf. Zhang et al. 2025). We retain this vernacular usage while recognizing that the capabilities and limitations of LLMs shape how such technologies function in family contexts. Understanding AI in family contexts requires moving beyond the view of technology as a neutral tool. Instead, AI constitutes a socio‐technical environment where human actors, digital systems, and family practices intertwine. Embedded in daily routines, AI both shapes and is shaped by parents' beliefs and practices; children's encounters with AI thus emerge through family dynamics rather than from technology alone (Livingstone and Helsper 2008; Nathanson 1999). Grounded in the Theory of Planned Behavior (Ajzen 1991) and parental mediation research, this study examines how parents interpret AI's role and how these interpretations translate into intended functions and mediation practices.

## 2 Literature Review

The rapid integration of AI into family and educational contexts has sparked increasing scholarly attention to how parents perceive, adopt, and regulate these technologies in early childhood. While AI is often framed as a promising tool to enhance children's learning and family life, it also raises complex questions about human interaction, safety, and developmental appropriateness (Glassman et al. 2021; Su 2025).

Previous studies indicate that parents generally recognize the potential benefits of AI tools in early education and care, particularly in home settings. Parents view AI as a means to enhance learning opportunities, support educational engagement, and relieve parental workload. For example, Glassman et al. (2021) found that parents believed AI could reduce technology interference and support structured learning, allowing children to engage productively with digital tools while limiting exposure to inappropriate content. Similarly, Zhang et al. (2025) documented parents' use of ChatGPT as a source of information, entertainment, and educational support, highlighting that AI tools can complement parental efforts by providing immediate responses and personalized content. Entenberg et al. (2023) further demonstrated that AI‐based chatbot interventions for parents can foster meaningful engagement and enhance parental efficacy, providing guidance for supporting children's socio‐emotional and cognitive development. Parents also perceived AI as capable of offering strategies, explanations, and structured practice, which could otherwise be limited by their own knowledge or time constraints (Khan et al. 2024; Shang et al. 2025).

Researchers consistently emphasize parents' concerns about human irreplaceability and developmental appropriateness. Physical safety, emotional security, and socio‐emotional learning are seen as domains where AI cannot substitute for human caregivers (Su 2025; Delaney and Chen 2025). Parents argued that AI could support repetitive tasks but could not replicate emotional attunement, guidance in creative expression, or nuanced social interaction, which are fundamental in early learning (Xiao et al. 2025). Age‐appropriateness also emerged as a key issue. Aslan et al. (2024) highlighted that parents are cautious about exposing young children to AI tools that may exceed their developmental stage. Similarly, Zhang et al. (2025) reported that parents preferred AI to supplement rather than replace parent‐led reading or learning activities, particularly emphasizing dialogue‐based engagement.

In addition to developmental concerns, privacy, safety, and ethics remain recurring themes in literature. Delaney and Chen (2025) recommended safeguarding children's personal data and avoiding commercialized AI tools. Glassman et al. (2021) and Su (2025) noted that parents worry about AI tools tracking children's behaviors, collecting sensitive data, or promoting marketing content, which could compromise children's well‐being. Similarly, Haley et al. (2024) found that parents of hospitalized children were cautious about AI in healthcare, a sentiment that extends to educational contexts—trustworthiness, transparency, and data security are prerequisites for parental acceptance.

Another critical dimension concerns parental mediation and active involvement. Studies consistently highlight that mediation strategies are central to ensuring safe and effective AI use at home. Shang et al. (2025) proposed a Learner‐AI‐Parent Collaboration Framework, emphasizing that parents actively guide interactions, monitor content, and scaffold children's learning. Xiao et al. (2025) found that parent‐led dialogic reading remained more effective than AI‐guided approaches unless parents mediated AI interactions to maintain engagement and comprehension. Glassman et al. (2021) also observed that parents exert control over screen time, device interfaces, and content selection to mitigate risks such as overuse, exposure to inappropriate material, or over‐gamification. Parents often use AI selectively for specific tasks, such as pronunciation practice, information queries, or creative storytelling, while maintaining core human‐mediated interactions for emotional and social learning.

Nevertheless, challenges in AI literacy and practical implementation remain. Su (2025) reported that while parents expressed positive attitudes toward AI, many lacked sufficient knowledge or confidence to integrate these tools effectively at home. Entenberg et al. (2023) and Zhang et al. (2025) emphasize that parent education and support are necessary for meaningful adoption, ensuring that AI enhances rather than undermines learning experiences. Khan et al. (2024) also pointed to concerns about assessment: while AI can support learning, parents worry about reliability in evaluating children's competencies, highlighting the need for AI tools to provide accurate feedback without replacing teachers' evaluative judgment.

Building on these themes, recent literature has begun to identify emerging trends and future directions. Parents increasingly see AI as a co‐participant in learning, complementing but not replacing human guidance (Shang et al. 2025; Zhang et al. 2025). Selective engagement is also evident, with parents mediating AI use based on developmental stage, interest, and task type. Hybrid models, which blend AI‐supported activities with parent‐led interactions, appear to maximize learning benefits while mitigating risks (Xiao et al. 2025; Su 2025). In addition, Delaney and Chen (2025) highlight the need for policy and guidance to help parents select safe, age‐appropriate, and non‐commercialized AI tools.

Taken together, literature underscores both opportunities and dilemmas in how families approach AI in early childhood. Although existing studies have examined parental attitudes toward digital technologies broadly, little is known about how parents' beliefs, their intended utility of AI, and their mediation practices interact in shaping children's AI use at home. Prior research often treats parental beliefs and parental mediation as separate constructs, overlooking the dynamic interplay between these constructs. Furthermore, most studies focus on screen‐time management or general digital literacy, leaving a gap in understanding how parents negotiate the unique opportunities and risks of AI in early childhood. By addressing this gap, the present study contributes to a more integrated framework for understanding parental decision‐making in the age of AI. The guiding research question is therefore: How do parents perceive and negotiate the role of AI in their young children's daily lives at home?

## 3 Method

### 3.1 Participants and Recruitment

Parents were purposefully selected according to three criteria: (a) prior AI use related to their child's education, (b) current nursery or kindergarten enrollment, and (c) willingness to provide detailed accounts. Recruitment involved posts in parent WeChat groups across nine Chinese cities, snowball sampling, and direct outreach to parents with existing contacts. Only parents reporting weekly use were included. Table 1 presents participant demographics and children's kindergarten contexts. Sixteen parents participated (14 mothers, 2 fathers), with children from nursery to senior kindergarten. Parent education ranged from bachelor's to master's degrees. Occupations included educators (kindergarten teachers, curriculum director, vocational teachers, university lecturer), professionals (lawyers, HR, engineer, AI product manager), and others (company employees, restaurant owner, foreign trade worker, athlete). Children were evenly distributed by gender (8 boys, 8 girls) and attended public (*n* = 7), private bilingual (*n* = 6), or international kindergartens (*n* = 3). Participants spanned nine Chinese cities, primarily Suzhou (*n* = 7).

**TABLE 1:** Participants' profiles (*N* = 16).

Participant code	Parent gender	Parent education	Parent occupation	Child age	Child gender	Kindergarten type	City
P01	Female	Master	University lecturer	Senior class	Male	Public	Ganzhou
P02	Female	Master	Lawyer	Senior class	Female	International	Suzhou
P03	Female	Bachelor	Kindergarten curriculum director	Senior class	Male	Public	Xuancheng
P04	Female	Bachelor	HR	Senior class	Female	Private bilingual	Bengbu
P05	Female	Bachelor	Kindergarten teacher	Senior class	Female	Public	Nanjing
P06	Female	Master	AI product manager	Middle class	Male	International	Suzhou
P07	Female	Master	Lawyer	Middle class	Male	Private bilingual	Suzhou
P08	Female	Bachelor	Vocational high school teacher	Middle class	Male	Public	Hefei
P09	Female	Bachelor	Vocational high school teacher	Middle class	Female	Public	Huainan
P10	Female	Bachelor	Company employee	Junior class	Female	Private bilingual	Suzhou
P11	Female	Master	Product engineer	Junior class	Male	Private bilingual	Suzhou
P12	Female	Bachelor	Restaurant owner	Junior class	Male	Private bilingual	Hefei
P13	Female	Bachelor	Kindergarten teacher	Nursery class	Female	Public	Suzhou
P14	Female	Master	Kindergarten teacher	Nursery class	Male	Private bilingual	Suzhou
P15	Male	Bachelor	Foreign trade company employee	Middle class	Female	International	Wuxi
P16	Male	Bachelor	Athlete	Senior class	Female	Public	Nantong

### 3.2 Data Collection

The first author conducted semi‐structured interviews in Chinese via WeChat video (35–75 min). Interviews were recorded, transcribed using 讯飞听见 (www.iflyrec.com), and selected excerpts were co‐translated into English by the first and second authors. Interview questions used “AI” broadly, as commonly understood, referring to technologies exhibiting intelligent responsiveness (smart speakers, learning apps, conversational agents, smart toys).

### 3.3 Data Analysis

We conducted thematic analysis (Braun et al. 2019; Naeem et al. 2023), involving iterative familiarization, coding, and category development through constant comparison. Three co‐authors engaged in reflexive discussion throughout the analytic process to refine codes and interpret emerging themes. During later stages of analysis, we observed that parents' accounts frequently moved from discussing their perspectives on AI to describing how they expected these technologies to be used in their children's lives, and subsequently to explaining how they regulated or guided children's interactions with these tools in practice. This recurring pattern informed our interpretation of relationships among the themes identified in the dataset. Building on this observation, we developed a conceptual framework that organizes these themes into a sequential relationship. To examine the robustness of this interpretation, we revisited the dataset to assess whether descriptions of mediation practices appeared independently of parents' broader perspectives on AI or their intended uses of it. No such instances were identified in the interviews, which reinforced our interpretation that these themes are conceptually linked. The framework is therefore presented as an analytic synthesis that organizes relationships among themes rather than as a causal or strictly linear model.

### 3.4 Researcher Positionality

The team comprised Chinese scholars in education, child development, and human‐computer interaction, sharing cultural and linguistic backgrounds with participants. The first author, the parent of a young child, conducted all interviews. This insider positionality facilitated rapport but required ongoing reflexivity to avoid imposing assumptions. Throughout analysis, we engaged in reflexive discussions, sought disconfirming evidence, and challenged assumptions. Co‐translation prioritized cultural meaning over literal equivalence, with disagreements resolved through consensus.

### 3.5 Ethical Considerations

Ethical approval was obtained from the Social Sciences Research Ethics Committee of United Arab Emirates University. All participants provided informed consent prior to participation, and confidentiality was assured throughout the research process.

## 4 Results

The analysis revealed a dynamic process through which families integrate AI into early education and care. During analysis, parents' accounts frequently moved from describing their perspectives on AI to explaining the functions they assigned to the technology and finally to outlining how they regulated children's interactions with it in everyday practice. This recurring pattern informed our interpretation of relationships among the themes identified in the dataset.

Accordingly, the findings are organized into three interconnected themes: parental beliefs, intended functions, and mediation strategies. Within each theme, several categories were identified that capture the ways parents make sense of and manage AI use in the home. All 16 parents contributed to each of the three major themes, though with varying emphasis across subthemes, with each subtheme endorsed by at least half of the sample. Table 2 presents the prevalence of each theme and subtheme across the sample.

**TABLE 2:** Prevalence of themes and subthemes across 16 parents.

Theme	Subtheme	Number of parents	Example parent codes
Theme 1: Parental beliefs	Importance of AI	16 (100%)	P01–P16
	Human irreplaceability	16 (100%)	P01–P16
	Principles for AI use	14 (87.5%)	P01–P11, P13–P15
Theme 2: Intended functions	Parental workload reliever	12 (75%)	P01–P05, P07–P11, P13–P14
	Parenting advisor	8 (50%)	P02, P05–P06, P08–P09, P13, P15–P16
	Entertainer	10 (62.5%)	P03, P05, P07–P14
	Game‐based learning	9 (56.25%)	P01, P03–P04, P06–P07, P09–P11, P15
Theme 3: Parental mediation	Presence control	8 (50%)	P02–P03, P06–P09, P11, P13
	Interface control	11 (68.75%)	P01–P08, P11, P13–P14
	Exposure control	13 (81.25%)	P01–P11, P13, P14
	Screen control	12 (75%)	P01–P11, P14
	Content control	10 (62.5%)	P01–P10

Parents first articulated core beliefs about AI, emphasizing its importance, the irreplaceability of human roles, and guiding principles for use. These beliefs shaped the functions they assigned to AI, including relieving parental workload, providing parenting advice, offering entertainment, and supporting game‐based learning. In turn, the intended functions informed how parents mediated AI use in practice, employing strategies such as controlling presence, interface, exposure, screen time, and content. Together, these themes and categories illustrate how parental values, expectations, and practices interact to frame children's encounters with AI in the home. Details of the categories are presented below, with each category illustrated by one or more quotes, and each quote attributed to the specific participant by including the participant code.

### 4.1 Theme 1: Parents' Beliefs

Parents spontaneously referenced multiple sources of their AI beliefs: personal experience (e.g., P06, AI product manager; P15, extensive work use), observations of children's engagement, peer conversations, media exposure, and educational values. P06's emphasis on creativity, aesthetic judgment, and questioning, for instance, reflected both professional knowledge and reflections on what human education must preserve. Parental beliefs thus synthesize multiple influences rather than deriving from any single source. Three subthemes emerged: AI's importance, human irreplaceability, and principles for responsible use.

#### 4.1.1 Category 1: Importance of AI

Parents universally acknowledged AI as transformative, stressing not replacement but adaptation. As P15 explained:

AI is extremely important. In every industry, it won't be AI that replaces people, but rather that those who cannot use AI will be left behind. AI is powerful and capable—I use it myself… so AI is not about simply substituting for humans, but about transforming work and eliminating those who fail to adapt.

This framing positions AI as an active participant in family life—a technology that responds, answers, and generates content, shaping parent–child interactions. Across accounts, AI emerged as a conversational partner, entertainer, and learning companion that children address directly. This agentic quality—initiating, responding, sustaining interaction—distinguishes AI from passive technologies and carries implications for family integration. Parents' proactive engagement reflects recognition that digital literacy is essential for both adults and children, framing AI as carrying broad societal implications beyond mere teaching.

#### 4.1.2 Category 2: Human Irreplaceability

Despite acknowledging AI's utility across applications (learning apps like Zebra Literacy, conversational agents like Doubao), parents insisted certain dimensions remain uniquely human: physical and emotional safety, core human capacities, and managing multiple children simultaneously. On safety and emotional care, P03 stated:

The most important aspect is safety. How could AI replace that? If you had an AI robot teacher, I don't think it would work… In this respect, AI would find it very difficult to substitute. At higher grades, maybe a lesson or two could one day be replaced. But for such young children, they need emotional care. Could a mother be replaced? Absolutely not. And a teacher in kindergarten is like the child's mother.

On core human capacities, P06 identified three that AI can never possess:

After using AI myself, I realized there are several things that are crucial for my child. The first is creativity… Second is aesthetic judgment… Third is expression. If you don't know how to ask questions, the answers AI gives you won't be ideal.

On group dynamics, Parent 04 observed, “Especially at the kindergarten stage, every child has a different personality and characteristics… There is no way AI could adapt to this.” These responses highlight AI's limitations in social and relational domains—interpersonal attunement, emotional reciprocity, and responding to subtle social cues (Zins et al. 2004). Safety, emotional care, creativity, and flexible responsiveness remain foundational human functions AI can only supplement, not replace.

#### 4.1.3 Category 3: Principles for AI Use

Parents articulated guiding principles: developmental appropriateness, privacy and safety, and avoidance of commercialization. On developmental appropriateness, P02 noted, “For young children, it's enough for them just to listen… I think for younger children, I still prefer that they read more books, more traditional reading.” P04 added, “At the preschool stage, learning is really secondary. Using AI for learning is just a bonus—if you don't have it, that's fine; if you do, parents think it's nice.”

On privacy, parents expressed acute awareness of data risks. P01 stated: “Usually when you register, you need to provide a name… If they asked for very specific details, like which kindergarten he attends, I would never provide that.” P09 added, “Things like recording a child's face, collecting personal information, full name, ID number, or other identifying details—I cannot accept that.”

On commercialization, parents resisted aggressive marketing and inappropriate content. P06 noted, “At first, I tried some AI learning software and robots, but later I was really driven crazy by their sales tactics… This is really a problem with commercialization in China; it affects our choices for children's education.” P12 described a poorly matched gift: “His grandfather bought it—I don't even know the brand… honestly my child can't even use it yet, because the content is at the elementary school level.”

Overall, Theme 1 demonstrates that parents adopt a balanced perspective toward AI: they value its transformative potential and practical benefits but insist on preserving human‐centric functions in early education and care. Safety, emotional care, creativity, and social responsiveness remain areas where AI is insufficient, while principles of age‐appropriate, privacy‐conscious, and non‐commercial use guide parents' adoption of AI tools.

### 4.2 Theme 2: AI's Intended Function in Early Education and Care

Parents described four intended functions for AI: workload relief, parenting guidance, entertainment, and game‐based learning.

#### 4.2.1 Category 1: Parental Workload Reliever

Parents viewed AI as alleviating repetitive tasks, freeing them for meaningful interactions. As P13 explained: “I think AI can help us with repetitive tasks, freeing up parents' time so we can spend it accompanying our children. Let AI focus on handling those repetitive jobs, whether for parents or teachers, so we can be liberated.”

“Repetitive jobs” included: answering repeated questions, reading stories on demand, providing pronunciation corrections, supplying on‐demand entertainment during chores, and monitoring simple instructional tasks. These tasks require patience and immediate responsiveness but not the creative, emotionally attuned engagement parents view as their irreplaceable contribution.

AI provided companionship when parental attention was limited. P05 described using Doubao: “He chats happily, asking Doubao questions or saying silly things—whatever he wants, Doubao responds… At least it keeps him company. It gives me 15 quiet minutes to cook dinner.” Parents valued AI's capacity to teach without emotional fatigue, as P15 said: “With AI, even if the child doesn't understand, it can patiently repeat things again and again. From this perspective, teaching without human emotions involved is actually a very good thing.” While parents appreciated this perceived patience and consistency, such views also raise questions about the absence of human interaction, through which children typically develop social and relational understanding during learning. AI also buffered against parental fatigue. P07 noted:

Many times, AI can free parents. Of course, human‐to‐human interaction is necessary, but if you have to interact with your child constantly, no one can handle it… Parents come home from work already exhausted—if they then have to interact for another two hours in the evening, honestly, they'd go crazy.

Parents identified specific tasks that AI could handle, such as:

- Responding to repetitive questions: “Doubao answers every time patiently.” [P05]
- On‐demand entertainment: “She asks the smart speaker for songs while I cook.” [P07]
- Independent practice: “The AI app corrects pronunciation when I can't.” [P11]
- Reading aloud: “The reading pen lets him follow books when I'm busy.” [P13]

This framing exists in tension with human irreplaceability. P07 reflected: “I feel guilty sometimes—like letting a machine do what I should. But I can't be ‘on’ all the time. The AI gives us a break, and then I have more energy to really play.” Parents thus navigate a pragmatic compromise between ideas of constant engagement and daily realities.

#### 4.2.2 Category 2: Parenting Advisor

Beyond workload relief, parents recognized AI as a source of knowledge and guidance. AI can provide access to broader experiences than individual parents might encounter: “It can search for other people's cases… It really does provide strategies—extracting the essence, discarding the dross, and summarizing from others' experiences. I think that's excellent. Our own circle is very limited” [P16]. Parents acknowledged that keeping up with the rapidly expanding body of knowledge in multiple domains is challenging. AI was seen as a compensatory mechanism, supplementing parents' educational resources:

Parents cannot always keep up with the times… But through AI, children can learn, and in that way AI compensates for the lack of educational information and resources that we as parents cannot provide. That's the most important aspect. [P08]

In this sense, AI functioned as an informational intermediary that parents could draw upon to support children's learning. At the same time, such reliance assumes that AI‐generated information is accurate and unbiased, raising questions about how parents evaluate or verify the guidance provided by these systems.

#### 4.2.3 Category 3: Entertainer

AI provided amusement through its distinct responsiveness. P11 observed: “When my child asks adults these kinds of questions, we often ignore him. But Doubao answers him seriously every time, and that makes him very happy. He asks absurd questions on purpose to see what AI will answer and finds it hilarious.”

This responsiveness—engaging whimsical inquiries without judgment or fatigue—generates interactions distinct from human responses. Children test AI's boundaries, finding delight in its mechanical yet consistent replies. AI becomes an interactive partner whose predictability creates novel play opportunities. Entertainment also fosters self‐efficacy. P03 noted:

When I let him use it on his own, he feels a real sense of accomplishment. He'll say, “Mom, did I draw all of these?” And I tell him, “Yes, look how carefully you're drawing now, you've learned so many skills, you can draw so much.”

AI's entertainment function thus supports engagement, curiosity, and emergent skills.

#### 4.2.4 Category 4: Game‐Based Learning

Finally, parents expressed caution regarding AI‐driven game‐based learning, noting the risk of over‐gamification and distraction from core learning objectives. P09 explained: “Hongen Literacy has too many games. At first, my child studied quite diligently and learned about 200 characters. But later he lost patience and just focused on playing games.” P10 added: “Many children end up reversing priorities. Later on, they only play the games for fun, but their actual character recognition gets weaker.” P15 noted: “Zebra Literacy also has too many games… Children get absorbed as if they were just playing games, and when you give them a real book afterward, they lose interest.” These reflections highlight the tension between engagement and educational efficacy, emphasizing the need for careful design and moderation.

Parents perceived AI as a supportive tool—relieving workload, providing guidance, entertaining—while cautioning against excessive gamification. These functions reflect a pragmatic approach: AI supplements, rather than replaces, human caregiving and educational roles.

### 4.3 Theme 3: Parental Mediation

Parents enacted five mediation strategies: presence, interface, exposure, screen, and content control.

#### 4.3.1 Category 1: Presence Control

Parents limited the presence of AI in their home learning environments to avoid redundancy with school instruction and to maintain a sense of authenticity in the child's experience. P11 stated: “We hadn't really looked into AI before, because we thought he already learns this at school, so we didn't want to push it at home.” P09 worried about highly AI‐integrated environments: “In our kindergarten, there isn't much AI, but I don't know about others. If I sent my child to a highly AI‐integrated school, I might worry that everything would be artificial, which I could not accept.” Parents ensured technology complements rather than dominates learning.

#### 4.3.2 Category 2: Interface Control

Parents actively managed the ways in which children accessed AI, emphasizing non‐visual, interactive modes that limit passive screen engagement. For instance, P03 described a screenless Tmall Genie: “He interacts via voice, asking about the weather, simple questions, or singing a song. That's enough. There are screens everywhere these days; it's better for children to have limited screen exposure.” Smartwatches were employed as interactive learning tools and for basic life skills, as P03 added:

He also uses a smartwatch to scan objects; the watch tells him what it is and describes it. It's like play. He also uses it to call his siblings, manage small amounts of money, buy things, and listen to stories on Ximalaya. The watch can do almost anything.

Occasional use of tablets was permitted, but primarily for guided reading or structured activities, with alternatives like reading pens to reduce passive screen time. P08 noted: “Many children have tablets, but ours doesn't use them much… He only uses the tablet occasionally, when he really wants to.”

#### 4.3.3 Category 3: Exposure Control

Parents monitored both the duration and frequency of AI use to prevent overreliance. They observed that without intervention, children might become excessively dependent. P13 articulated: “Although he liked it, I didn't let him keep using it continuously. He used it for a while, then stopped relying on it. There was a period when he was very dependent, wanting to use Zebra daily, sometimes for long periods.” P08 echoed, “If you don't control it, he could chat with it from morning until night.” Exposure control reflects a proactive approach to regulate children's engagement with AI, ensuring it remains supplemental rather than central to daily activities.

#### 4.3.4 Category 4: Screen Control

Screen time was explicitly managed, with parents favoring smart speakers over televisions and limiting sessions to short intervals. P14 elaborated:

We don't have a TV, because we worry about screen time and children's eyes. But we have a Tmall Genie smart speaker, and my son likes interacting with it every day. We try to keep it under half an hour daily. We don't strictly track time; a Zebra Thinking session is about ten minutes, and then we turn it off so his eyes can rest.

Some parents relied on natural activity rhythms to balance screen use, pairing AI interaction with outdoor play. P07 said, “I think it's fine. Children enjoy it but won't stare endlessly. We also take him outside to play every day after meals with friends at the nearby mall, so it balances out.” These practices indicate that parents integrated AI into children's routines while maintaining holistic attention to health and activity.

#### 4.3.5 Category 5: Content Control

Parents expressed concern about unfiltered content accessed via voice‐controlled devices. P04 described:

With home AI devices, children use smart devices that can be voice‐controlled, so they can interact without knowing how to read. Children cannot filter information, so they may be exposed to inappropriate content. You never know where they hear certain things, and they may hear inappropriate words.

Content control reflects parents' recognition of children's developmental limitations in evaluating information, reinforcing the need for guided and supervised AI use.

Overall, Theme 3 illustrates that parents adopt multi‐layered mediation strategies to regulate AI in home learning environments. By controlling presence, interface, exposure, screen time, and content, parents aim to maximize AI's educational and entertainment benefits while mitigating risks related to overuse, overreliance, and inappropriate content. These findings emphasize the active role of parents in shaping safe, developmentally appropriate AI experiences in early education and care.

### 4.4 The Sequential Framework

**FIGURE 1:**
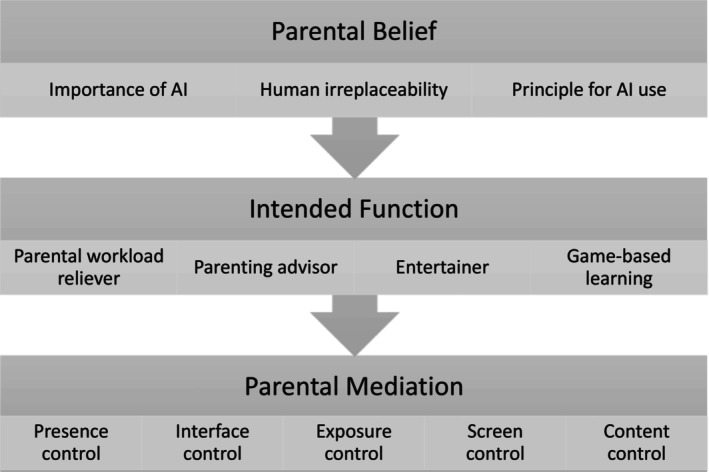
The process of family AI use in early education and care.

While themes were derived independently, their relationship points to a broader process. We observed a conceptual logic organized into a sequential framework: parents' beliefs provide foundations for the functions they assign AI, and these functions guide mediation strategies. This represents our analytic construction—a way of making sense of the data—rather than a claim that parents consciously follow this sequence in real time. This logic aligns with Theory of Planned Behavior (Ajzen 1991), which posits that beliefs and attitudes shape intentions, and intentions subsequently guide behaviors. It is further substantiated by Parental Mediation Theory (Livingstone and Helsper 2008; Nathanson 1999), which posits that perceptions of media determine regulatory strategies. Thus, beliefs, intended functions, and mediation strategies form a coherent process explaining how families structure children's AI encounters. Figure 1 illustrates this framework.

We present this as unidirectional for conceptual clarity but recognize family processes are rarely linear. Beliefs may shift through experience; mediation challenges may reshape beliefs; successful mediation may reveal new functions. The framework is an idealized conceptual pathway, acknowledging that real families move through these phases iteratively and recursively. Longitudinal research could examine these feedback loops empirically.

### 4.5 AI Tools Referenced by Parents

The following AI tools were mentioned by parents during interviews:

- Doubao: Conversational AI agent (app/smart speaker) for storytelling, questions, conversation.
- Tmall Genie: Smart speaker for music, stories, information queries.
- Zebra Literacy (Zebra Thinking): AI literacy app with gamified character learning.
- Hongen Literacy: AI app for Chinese character recognition with game elements.
- Ximalaya: Audio platform with AI‐recommended stories and content.
- Smartwatches: Wearables with AI functions (object recognition, communication, payment).

## 5 Discussion

This study sheds light on how Chinese parents make sense of AI in early education, moving beyond acceptance/rejection toward a nuanced framework of beliefs, functions, and mediation. Parents framed AI as supplementary—enhancing rather than displacing human interaction. Their accounts reflect dual orientation: optimism about AI's potential for learning, convenience, and engagement, alongside caution about safeguarding children's well‐being, creativity, and moral development. These perspectives translated into concrete mediation strategies, positioning parents as active gatekeepers regulating AI's extent, quality, and purposes at home.

### 5.1 The Process Framework of Family AI Use in Early Education and Care

The Process Framework integrates parents' beliefs, intended functions, and mediation practices into a sequential structure, advancing scholarship by revealing family AI decision‐making as a coherent process rather than discrete actions. While prior research has examined parental beliefs (Aslan et al. 2024; Glassman et al. 2021; Haley et al. 2024; Su 2025), perceived functions (Entenberg et al. 2023; Khan et al. 2024; Shang et al. 2025; Xiao et al. 2025), and mediation (Livingstone and Helsper 2008; Nathanson 1999) separately, this framework bridges these strands into a unified conceptual pathway explaining how families move from evaluating AI to shaping children's engagement.

Grounded in inductive analysis, the framework serves as a conceptual tool for understanding parental decision‐making. Its cross‐disciplinary scope—spanning family studies, early education, and technology—informs scholarly and practical discussions on family engagement, parenting practices, child development, and negotiation of family roles. Generated from urban Chinese data, the framework's components (beliefs, functions, mediation) may also be relevant in other cultural contexts, although their specific forms and meanings are likely to differ. Chinese parents' emphasis on academic achievement and structured learning, for instance, may influence AI's perceived role, while commercialization concerns reflect local market conditions. Cross‐cultural research is needed to examine manifestation across settings. The framework is not exhaustive but offers a springboard for developing tailored frameworks for specific cultural, technological, or developmental contexts.

### 5.2 Parental Mediation as a Family Regulatory Process

Parental mediation of AI parallels long‐standing strategies like co‐viewing, restrictive mediation, and active discussion (Livingstone and Helsper 2008; Nathanson 1999). However, AI introduces new dimensions: parents now regulate not only content and screen time but also algorithmic influence, privacy, and commercial pressures. This expands mediation theory into contexts where regulation extends beyond visible media exposure to hidden data flows and automated personalization.

The mediation strategies identified reflect collective practices across the sample, not individual families. Families prioritize different control areas based on values, experiences, and contexts, underscoring the need for research into determinants of mediation choices. These strategies should not serve as evaluative criteria for specific families but rather formalize our conceptual understanding of AI mediation at home.

This mediation constitutes a family regulatory process: parents collectively structure, monitor, and guide children's AI engagement, balancing autonomy, safety, and learning. Such regulation helps children navigate digital environments safely, promotes responsible technology use, and establishes shared family routines and expectations. It further contributes to family strength by fostering communication, reinforcing parental roles, and aligning family values and practices. Families actively engaged in such regulation may experience greater cohesion—emotional bonding among members (Olson 2000)—and shared understanding—mutual awareness of expectations, values, and routines (Epstein et al. 1993)—underscoring the broader developmental significance of parental mediation in AI contexts.

### 5.3 AI as a Co‐Participant in Family Interaction

Our findings suggest AI functions as an active co‐participant in family dynamics. Parents described AI not as a neutral device but as an interlocutor children address directly, that generates satisfying responses, and that shapes interactional tone. When a child poses an “absurd question on purpose” and both parent and child share laughter at the outcome, AI becomes a catalyst for joint attention and shared positive affect—actively participating in constructing family experiences rather than merely delivering content.

This observation aligns with sociomaterial perspectives (Barad 2007; Orlikowski 2007), which conceptualize technology and human practice as mutually constituting. AI's agency emerges through entanglement with family routines, parental values, and children's curiosity. Boundaries blur: parents guide children's use, but AI's responses also guide parents' next moves—sustaining interaction, suggesting activities, or prompting intervention.

While parental mediation highlights parents' regulatory role, our findings suggest mediation is not unidirectional. AI systems actively shape family interactions, generating responses, entertainment, and prompts that influence how parents and children engage. Parents described shared amusement when children posed unusual questions to AI and laughed at responses. Mediation thus operates relationally: parents guide children's AI use while AI simultaneously shapes household interactions (Livingstone and Helsper 2008; Nathanson 1999), resonating with sociomaterial perspectives on jointly shaped social practices (Barad 2007).

Recognizing AI's agentic role extends parental mediation theory. Traditional frameworks assume parents act upon passive media. In AI‐mediated environments, parents negotiate with technology that talks back, learns, and adapts. This demands new literacy: understanding not only how to limit or filter but how to engage with interactive systems whose responses are neither fully predictable nor controllable.

### 5.4 Paradoxes of AI‐Mediated Family Life

This study reveals several paradoxes in how families experience AI. First, AI functions simultaneously as connection and disruption. Parents described shared amusement and reduced stress alongside concerns about distraction, overuse, and constant mediation. The same technology providing “15 quiet minutes” to cook dinner can, uncontrolled, lead to children “chatting from morning until night”. AI's effects are not inherent but depend on integration and regulation.

Second, AI both reduces and expands parental labor. Parents value workload relief yet face new demands: monitoring content, managing screen time, evaluating privacy risks, resisting commercial pressures. As P06 noted, “I have to be more vigilant now—there's just more to keep track of.”

Third, parents navigate competing commercial and educational pressures. Seeking developmentally appropriate tools, they encounter aggressive marketing, distracting gamification, and data collection conflicting with privacy values. These commercial dimensions actively shape AI's family role; mediation strategies—content control, avoiding commercialized tools—respond to this tension.

These paradoxes highlight that AI integration involves ongoing negotiation of competing values, needs, and constraints. The Process Framework captures the sequential logic of parental decision‐making but operates within these tensions rather than resolving them.

### 5.5 AI Capabilities and Parental Awareness

While parents largely perceived AI as beneficial, these views raise broader questions. Some parents valued teaching without human emotions; however, child development research emphasizes learning as embedded in social interaction, through which children develop relational and socio‐emotional competencies. Reliance on AI may complement learning but could reduce human interaction if used uncritically. Moreover, AI systems are known to generate inaccurate, biased, or inappropriate content (Maslej et al. 2023). Parents did not raise concerns about factual reliability, suggesting trust in these systems, limited awareness of their limitations, or absence of critical incidents. This absence is noteworthy given children's vulnerability to accepting AI‐generated information uncritically. Parents' characterization of AI as “parenting advisor” and “informational intermediary” therefore warrants caution. AI accuracy varies across domains and platforms; parents may benefit from guidance on evaluating AI‐generated information alongside their children.

Similarly, parents' appreciation of AI's patience in repetitive tasks raises questions about the value of human persistence in learning. While AI's endless patience may seem advantageous, research suggests frustration, struggle, and persistence through difficulty are important developmental experiences (Kapur 2016). When AI eliminates these struggles, children may miss opportunities to develop resilience and problem‐solving skills. Research is needed to examine whether AI‐mediated learning inadvertently reduces productive struggle and how parents navigate this trade‐off.

### 5.6 Intergenerational Dynamics of AI Adoption

Parents actively interpret AI's implications for family roles, authority, and relational balance. By positioning AI as supplementary, they reinforce their irreplaceable roles as nurturers, moral guides, and emotional anchors, even as technology potentially disrupts traditional hierarchies of learning and caregiving.

AI creates complex intergenerational dynamics. As “workload reliever”, it allows parents to manage household responsibilities while children engage with learning; as “entertainer”, it generates shared joy and amusement. Both parent and child experience simultaneous positive emotions, reducing tension, facilitating cooperation, and strengthening emotional climate. One parent noted, “we both get a break and laugh together—it makes the day smoother”, illustrating technology as a mediator of family harmony rather than conflict.

These dynamics are family‐specific, distinct from classrooms where interactions are structured around pedagogical goals, standardized curricula, and teacher authority. At home, AI‐mediated learning allows flexible co‐engagement, reciprocal attention, and shared emotional regulation, highlighting the interplay between learning, caregiving, and affect.

Findings suggest reconceptualizing parental involvement, particularly parent–child interaction and family literacy. AI not only mediates knowledge acquisition but co‐constructs emotional, moral, and practical experiences shaping intergenerational learning. This challenges traditional frameworks of learning as unidirectional knowledge transfer, underscoring technology's potential to create bidirectional, emotionally rich, relationally negotiated learning environments within families—positioning the household as a unique site of technologically mediated intergenerational learning.

### 5.7 Implications for Family Policy and Support Systems, and Family Therapy

The findings highlight the need for family‐centered policies and guidance that account not only for safe and ethical AI use but also for the broader relational and emotional dynamics within households. Parents' concerns about privacy, commercialization, and responsible AI use underscore the importance of regulatory frameworks that protect families while enabling educational innovation.

Family support services and educational programs should go beyond technical guidance to equip parents with the skills to critically evaluate AI, mediate its use, and intentionally shape intergenerational learning experiences. This includes supporting parents in leveraging AI as a tool that can reduce stress, foster positive interactions, and strengthen family cohesion, rather than simply managing content or screen time. Programs could provide strategies for balancing parental authority with child autonomy, integrating AI into daily routines, and promoting shared emotional and educational experiences.

Ultimately, policy and support systems should recognize that AI‐mediated learning in the home is not just a technological issue but a family regulatory process. Supporting families in this broader sense can enhance not only children's learning outcomes but also family functioning, emotional wellbeing, and intergenerational bonds, positioning the household as a unique site of technologically mediated learning and relational development.

The Process Framework also offers practical insights for family therapists. As technology becomes increasingly embedded in family life, therapists encounter families navigating conflicts over digital media use and child autonomy. Information communication technologies shape family dynamics and intimacy (Bacigalupe and Lambe 2011), and our findings extend this understanding to AI in early childhood, showing that AI functions as a co‐participant in family interaction. Therapists can use the Framework to assess whether a family's AI‐related difficulties originate in parental beliefs, intended functions, or mediation strategies. Technology‐mediated contexts require therapists to develop digital competencies (Aviram and Nadan 2022). Therapists should routinely inquire about AI use at home, normalize both enthusiasm and concern, and help families develop flexible, value‐congruent mediation practices, including co‐creating shared rules for AI use.

### 5.8 Limitations and Future Direction

Several limitations should be acknowledged. First, the study focused exclusively on urban Chinese parents, limiting generalizability to other cultural, socio‐economic, or rural contexts. The findings reflect a specific setting where educational technology adoption is widespread, family investment in early learning is high, and parents navigate unique commercial and policy environments. Relatedly, the sample may overrepresent parents with higher technological literacy, potentially biasing perspectives toward more positive views of AI adoption. The framework requires empirical validation across diverse populations to assess its applicability in varying contexts. Second, parents' characterization of AI as “informational intermediary” should be interpreted within the technical constraints of current LLMs, which generate inaccurate information or “hallucinations” (Maslej et al. 2023). Parents did not raise concerns about factual reliability, suggesting trust in these systems or limited awareness. Whether children's exposure to inaccuracies carries developmental implications remains an open question. Third, this study captured only parental perspectives, not children's own experiences or agency. While parents serve as primary gatekeepers, children may experience AI differently. Young children are not passive recipients but active interpreters who may resist, subvert, or creatively appropriate AI technologies.

Future research could address these limitations through several lines of inquiry. First, cross‐cultural studies are needed to examine how AI adoption and parental mediation vary across socio‐cultural, economic, and rural contexts, extending beyond the urban Chinese setting of the present study. Recruiting more diverse samples would also help assess whether parents with varying levels of technological literacy hold different perspectives on AI in early childhood. Second, given parents' trust in AI‐generated information, research should investigate how families evaluate the accuracy and reliability of AI responses, and whether children's exposure to inaccuracies carries developmental implications. This includes examining parents' awareness of LLM limitations and how they might be supported in critically assessing AI‐generated content alongside their children. Third, future studies should incorporate children's voices directly through child‐centered methods such as observations, participatory activities, or developmentally adapted interviews. Understanding how young children experience, interpret, and appropriate AI technologies will complement parental accounts and provide a fuller picture of AI‐mediated family life.

## 6 Conclusion

This study shows that Chinese parents' use of AI in early education and care reflects a sequential and relational process: beliefs about AI shape its expected functions, which guide mediation strategies at home. Parents treat AI as a supportive but supplementary tool, balancing benefits and risks while maintaining their irreplaceable role as educators and emotional anchors. AI‐mediated learning functions as a family regulatory process, fostering positive intergenerational interactions, reducing stress, and strengthening family cohesion—dynamics rarely seen in non‐family settings. These findings highlight the pivotal gatekeeping role of parents, extend theories of parent–child interaction and family literacy, and provide a conceptual framework to guide the design, integration, and governance of AI in early childhood contexts.

## Funding

This study was funded by United Arab Emirates University (12R258).

## Ethics Statement

The study was approved by the Social Sciences Research Ethics Committee of United Arab Emirates University.

## Conflicts of Interest

The authors declare no conflicts of interest.

## Data Availability

The data that support the findings of this study are available at https://doi.org/10.5281/zenodo.16961734.
